# Curcumin and Solid Lipid Curcumin Particles Induce Autophagy, but Inhibit Mitophagy and the PI3K-Akt/mTOR Pathway in Cultured Glioblastoma Cells

**DOI:** 10.3390/ijms20020399

**Published:** 2019-01-18

**Authors:** Panchanan Maiti, Jason Scott, Dipanwita Sengupta, Abeer Al-Gharaibeh, Gary L. Dunbar

**Affiliations:** 1 Field Neurosciences Institute Laboratory for Restorative Neurology, Central Michigan University, Mt. Pleasant, MI 48859, USA; to.abeer@gmail.com; 2 Program in Neuroscience, Central Michigan University, Mt. Pleasant, MI 48859, USA; 3 Department of Psychology, Central Michigan University, Mt. Pleasant, MI 48859, USA; 4 Field Neurosciences Institute, St. Mary’s of Michigan, Saginaw, MI 48604, USA; Dipanwita.Sengupta@ascension.org; 5 Department of Biology, Saginaw Valley State University, Saginaw, MI 48710, USA; jascott1@svsu.edu; 6 Brain Research Laboratory, Saginaw Valley State University, Saginaw, MI 48710, USA

**Keywords:** glioblastoma multiforme, autophagy, mitophagy, curcumin, chaperone-mediated autophagy, Akt/mTOR signaling, transmission electron microscopy

## Abstract

Autophagy and the (PI3K-Akt/mTOR) signaling pathway play significant roles in glioblastoma multiforme (GBM) cell death and survival. Curcumin (Cur) has been reported to prevent several cancers, including GBM. However, the poor solubility and limited bioavailability of natural Cur limits its application in preventing GBM growth. Previously, we have shown the greater apoptotic and anti-carcinogenic effects of solid lipid Cur particles (SLCP) than natural Cur in cultured GBM cells. Here, we compared the autophagic responses on cultured U-87MG, GL261, F98, C6-glioma, and N2a cells after treatment with Cur or SLCP (25 µM for 24 h). Different autophagy, mitophagy, and chaperone-mediated autophagy (CMA) markers, along with the PI3K-AKkt/mTOR signaling pathway, and the number of autophagy vacuoles were investigated after treatment with Cur and or SLCP. We observed increased levels of autophagy and decreased levels of mitophagy markers, along with inhibition of the PI3K-Akt/mTOR pathway after treatments with Cur or SLCP. Cell survival markers were downregulated, and cell death markers were upregulated after these treatments. We found greater effects in the case of SCLP-treated cells in comparison to Cur. Given that fewer effects were observed on C-6 glioma and N2a cells. Our results suggest that SLCP could be a safe and effective means of therapeutically modulating autophagy in GBM cells.

## 1. Introduction

According to the World Health Organization (WHO), glioblastoma multiforme (GBM) is one of the deadliest and most aggressive brain cancers, affecting millions of people world-wide. Histopathological analysis revealed that brain tumors account for 85% to 90% of all primary Central nervous system (CNS) tumors, and about 70% to 80% are of glial cell origins. Whereas among all primary brain tumors only 15% are GBM [1]. Importantly, most GBM patients survive on average for only 15 to 20 months following initial diagnosis [2]. Despite current neurosurgical, radiotherapy, and chemotherapeutic advancement, the GBM growth and proliferation cannot be effectively controlled. The most potent chemotherapeutic drug use to treat GBM is temozolomide (Temodar, TMZ), but resistance to TMZ limits its effectiveness. Moreover, neuroinflammation also increases after treatment of TMZ, making the development of alternative therapies critically important. In this context, several investigators have studied the anti-cancer and anti-inflammatory effects of curcumin (Cur) in human malignancies, including those found in various tissues, such as breast, prostate, colon, liver, and brain [3,4].

Several anti-cancer drugs have been tested to prevent GBM cell growth and metastasis [4]. Many of these drugs kill GBM cells by inducing apoptosis, autophagic cell death, or necrosis, whereas dysregulation of these major pathways promotes cancer development [5]. Although apoptosis is the most common form of programmed cell death (PCD), autophagic cell death also has significant roles in tumorigenesis [5]. It includes macroautophagy, microautophagy, and chaperones-mediated autophagy (CMA), which are highly conserved cellular-debris disposal mechanisms by which cellular organelles, misfolded protein aggregates, and pathogens or toxins are degraded through fusion of the resulting autophagosomes with lysosomes [6,7,8]. The beneficial effects of autophagy have been observed with anti-cancer drugs after their treatments in GBM cells, which may either induce or bypass the apoptotic pathway, depending on cellular stress [9]. Several experimental results from animal studies and cell culture studies have demonstrated that induction of autophagy or type-II PCD can induce or inhibit type-I PCD or apoptosis [10], which suggests that they are inter-linked for cell death and survival [11]. In addition, cancer cell growth and proliferation are also controlled by the phosphatidylinositol 3-kinase (PI3K)/Akt/mammalian target of rapamycin (mTOR, also known as the mechanistic target of rapamycin and FK506-binding protein 12-rapamycin-associated protein 1) sensitive mTOR-complex (PI3K-Akt/mTOR) pathway, which has inhibitory roles on the autophagic pathway. The Akt/mTOR pathway plays significant roles in the regulation of autophagy, as well as cancer cell growth and proliferation; inhibition of this pathway has activatory roles in the autophagy pathway and inhibitory roles on cancer cell proliferation. Therefore, targeting autophagy by inhibiting PI3K-Akt/mTOR might be a potent strategy to inhibit GBM growth and proliferation [12].

Curcumin (Cur), is the most active natural polyphenol present in the turmeric root of the herb, *Curcuma longa* [13]. For a long time, it has been known to function as a potent inhibitor of tumor growth, proliferation, invasion, angiogenesis, and metastasis. Cur has been applied for several cancer therapies, including GBM [14]. It can attenuate cancer growth by increasing oxidative stress, disrupting PI3k-Akt/mTOR signaling and induction of apoptosis, but it requires higher amounts to be effective against cancer cells [15]. Unfortunately, poor solubility and instability in physiological fluids limits its therapeutic application for targeting GBM [16,17]. Although various lipidated and nanotechnological approaches of Cur formulations have been shown to increase its solubility and bio-availability [15], none of these produce optimal levels. Recently, solid lipid particles (SLPs), conjugated with Cur (SLCPs), have been characterized by our laboratory [15,18,19] and those of others to increase Cur solubility, stability, and bioavailability [20,21,22,23,24,25], when tested in an in vitro model of GBM, as well as animal models and clinical trials of Alzheimer’s disease [26,27].

Previously, we have reported that SLCPs induce a greater number of apoptotic deaths than natural Cur in U-87MG [19]. In the present study, we have designed the experiments to compare the autophagy mechanism, including mitophagy and the PI3K-Akt/mTOR pathway (which is one of the modulators of the autophagy pathway) in vitro, using GBM cells derived from human (U-87MG), mouse (GL261), and rat (F98) origins, their respective rat glial tumor cells (C6-glioma), and mouse neuroblastoma cells (N2a cells) after treatment with Cur and/or SLCP. Our results suggest that SLCP induced autophagy markers greater than natural Cur, as well as the inhibition of mitophagy and the significant disruption of the PI3K-Akt/mTOR pathway in all three GBM cells, without significant effects on C6-glioma and N2a cells.

## 2. Results

In this study, we have compared the levels of autophagy, including mitophagy markers and the PI3k-Akt/mTOR signaling pathway in cultured GBM cells after treatment with SLCP and or Cur.

### 2.1. SLCP Induced Autophagy Greater than Natural Cur in Different GBM Cells

**Figure 1:**
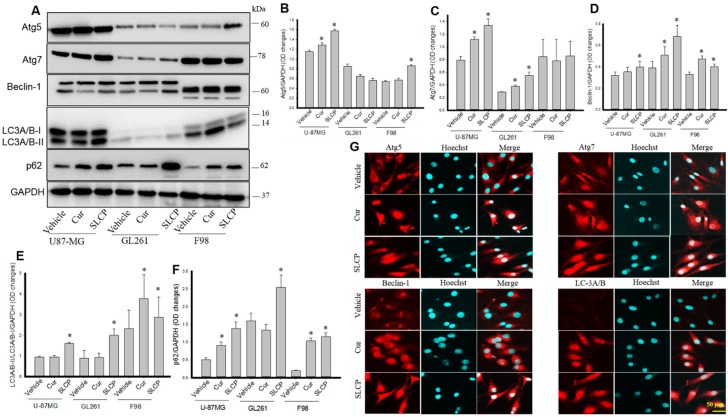
Changes of autophagy markers in GBM cells after treatment with Cur and or SLCP. (**A**–**F**): U-87MG, GL261, and F98 cells were treated with either Cur or SLCP (25 µM) for 24-h and then Western blots and immunocytochemistry (ICC) were performed. The Western blots data showed that there were significant increased levels of Atg5, Atg7, Beclin-1, LC3A/B, and p62 after treatment with SLCP and/or Cur. Values are represented as mean ± standard error of mean (SEM) from three independent observations. * *p* < 0.05 in comparison to the respective vehicle-treated group. (**G**): Immunocytochemisty (ICC) revealed apparent increases in ICC intensity of Atg5, Atg7, Beclin-1, and LC3/A/B in SLCP- and Cur-treated U-87MG cells in comparison to vehicle-treated cells. Scale bar indicates 50 µm and is applicable to all images.

**Figure 2:**
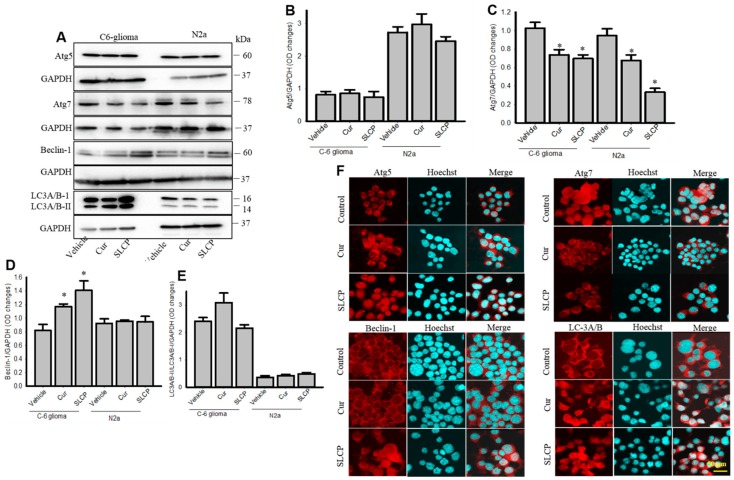
Changes of autophagy markers in C6-glioma and N2a cells after treatment with SLCP and Cur. C6-glima and N2a cells were treated with either Cur or SLCP (25 µM) for 24-h prior to performing Western blots and ICC. (**A**–**E**): The Western blots analyses revealed that there were no significant changes of autophagy markers following treatment, except Atg7 levels were significantly decreased in both cell lines (**C**) and Beclin-1 levels were increased in C6-glioma (**D**). Values are represented as mean ± SEM from three independent observations. * *p* < 0.05 in comparison to the respective vehicle-treated cells. (**F**): ICC of autophagy markers in N2a cells showed that there were no major changes of the immunofluorescent signal for these parameters. Scale bar indicates 50 µm and is applicable to all images.

We have investigated different autophagy markers, such as Atg5, Atg7, Beclin-1, LC3A/B, and p62, from all three GBM cell lines (U-87MG, GL261, and F98), and from C6-glioma and N2a cells. We observed that the Atg5 level was significantly increased (*p* < 0.05) in U-87MG and F98 cells, but not in GL261 after treatment with Cur and or SLCP in comparison to vehicle-treated groups (Figure 1A,B). Similarly, we found a significant increase (*p* < 0.01) in levels of Atg7 after Cur and or SLCP treatment in U-87MG and GL261, but not in F98 cells, in comparison to the vehicle-treated group (Figure 2A,C). Furthermore, the Beclin-1 level was also significantly increased (*p* < 0.05) in all three GBM cells after treatment with Cur or SLCP in comparison to the vehicle group (Figure 1A,D). We also observed that the ratio of LC3A/B-II/LC3A/B-I was significantly increased by Cur and or SLCP treatment in all three GBM cells lines in comparison to vehicle-treated cells (Figure 1A,D). SLCP-treated cells had more changes in autophagic markers, overall, than did Cur-treated cells. Similar to the Western blot data, the immunofluorescence intensity of Atg5, Atg7, Beclin-1, and LC3A/B all tended to increase in U-87MG cells after treatment with Cur and or SLCP, in comparison to vehicle-treated cells (Figure 1G).

### 2.2. Cur and or SLCP Treatment Has Little Influence on Autophagy Pathways in Rat Glial Tumor Cells (C6-Glioma) and Mouse Neuroblatsoma (N2a) Cells

We did not observe any significant changes of Atg5 (Figure 2A,B) and LC3A/B levels (Figure 2A,E) after treatment with Cur and or SLCP treatment. In addition, we observed very little differences of Atg7 levels in C6-glioma cells, and there was a decrease in N2a cells (Figure 2A,C) after Cur and or SLCP treatment. In contrast, Beclin-1 was increased in C6-glioma, but not in N2a cells (Figure 2A,D) after Cur and or SCLP treatment. The ICC of Atg5, Atg7, Beclin-1, and LC3A/B in U-87MG also showed comparable results, as shown in the Western blots after Cur and/or SLCP treatment (Figure 2F).

### 2.3. SLCP Inhibits Mitophagy Markers More than Cur in GBM Cells.

**Figure 3:**
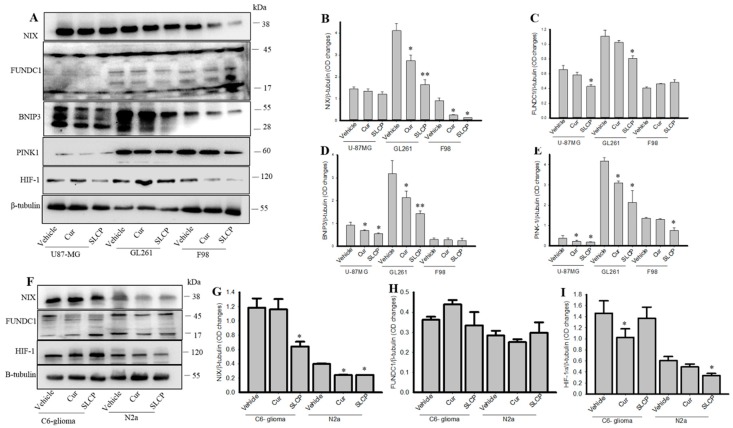
Mitophagy markers were down-regulated more in GBM cells by SLCP than by Cur. U-87MG, GL261, F98, C6-glioma, and N2a cells were treated with either Cur or SLCP (25 µM for 24-h) prior to Western blots analyses. The levels of BNIP3L/NIX, FUNDC1, BNIP3, PINK-1, and HIF-1α were significantly down-regulated (*p* < 0.05) after treatment of SLCP and or Cur in all three GBM cell lines (**A**–**E**), and SLCP showed greater decreases of these parameters than did Cur. Both NIX and HIF-1α were decreased in C6-glioma and N2a cells, but not FUNDC1 (**F**–**I**). Values are represented as mean ± SEM from three independent observations. * *p* < 0.05 and ** *p* < 0.01, in comparison to the respective vehicle-treated cells.

We also investigated the most important mitophagy markers, such as BNIP3L/NIX, FUNDC1, BNIP3, PINK-1, and HIF-1α, by Western blots. We observed that BNIP3L/NIX was significantly decreased in GL261 and F98 cells (*p* < 0.05) and there was a trend of reduction of this protein in U-87MG after treatment with Cur and SLCP, in comparison to the vehicle-treated group (Figure 3A,B). Similarly, the FUNDC1 level was also significantly decreased in SLCP-treated cells greater than the Cur treated and vehicle-treated group (Figure 3A,C). In addition, BNIP3, PINK-1, and HIF-1α levels were also significantly decreased (*p* <0.05) in Cur and or SLCP-treated cells in comparison to the vehicle-treated group (Figure 3A,D,E). Whereas, we found a significant decrease of BNIP3L/NIX and HIF-1α in C6-glioma and N2a cells after treatment with Cur and or SLCP (Figure 3F–I), whereas no significant changes were observed in the case of FUNDC1 protein in both the cell lines (Figure 3F,H).

### 2.4. SLCP Inhibits PI3K-Akt/mTOR Pathway Activity in GBM Cells More than Cur does in GBM

**Figure 4:**
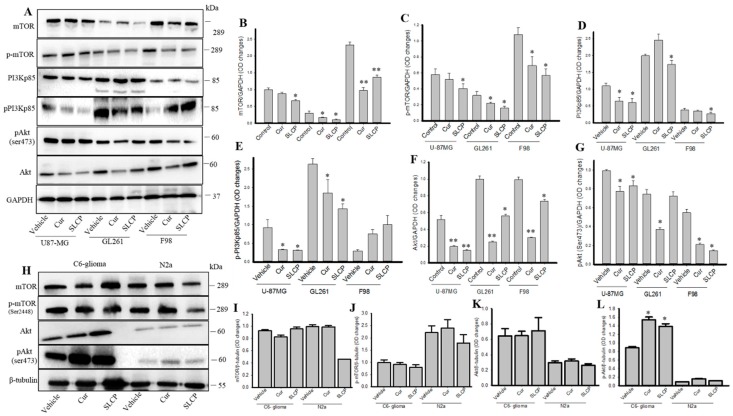
SLCP inhibited the PI3K-Akt/mTOR pathway greater than Cur in cultured GBM cells. U-87MG, GL261, F98, C6-glioma, and N2a cells were treated with either Cur or SLCP (25 µM for 24-h) prior to Western blots and ICC. (**A**–**G**): Western blots analyses revealed that PI3Kp85, pPI3Kp85, total Akt, p-Akt (Ser473), mTOR, and p-mTOR levels were significantly decreased in all three GBM cells after treatment with Cur and or SLCP, in comparison to the respective vehicle-treated cells. These parameters were unaltered in C6-glioma and or N2a cells (**H**–**L**), except that pAkt was significantly increased in C6-glioma after the treatment. Values are represented as mean ± SEM from three independent observations. * *p* < 0.05 and ** *p* < 0.01 in comparison to their respective vehicle-treated cells.

We observed that PI3Kp85, phosphoPI3Kp85, total Akt, p-AKT, mTOR, and p-mTOR levels were significantly decreased in U-87MG, GL261, and F98 cell lines after treatment with Cur and or SLCP (Figure 4A–D). SLCP showed greater inhibition of the PI3k-Akt/mTOR pathway than Cur. Whereas in the case of C6-glioma and N2a cells, this pathway was mostly unaltered (Figure 4G–K).

### 2.5. Cur and or SLCP Treatment Inhibited Chaperone-Mediated Autophagy (CMA) Markers in GBM Cells.

**Figure 5:**
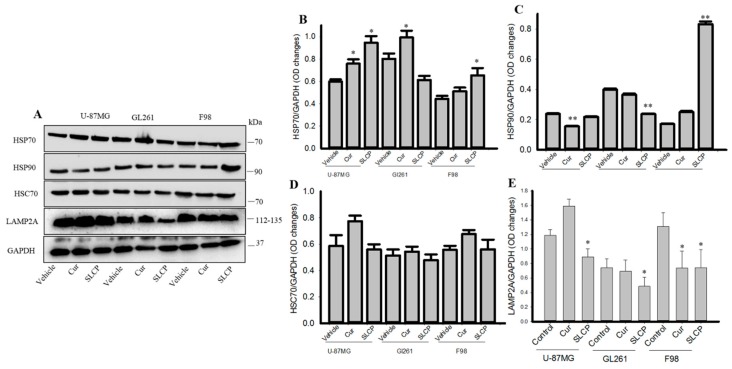
Chaperone-mediated autophagy markers were inhibited in GBM cells after SLCP or Cur treatment. U-87MG, GL261, F98, C6-glioma, and N2a cells were treated with either Cur or SLCP (25 μM for 24-h) prior to Western blots. HSC70 levels were unaltered by SLCP or Cur treatment, while LAMP2A levels were significantly decreased in all the GBM cell lines (**A**,**D**,**E**). There were significant increases in levels of HSP70 (**A**,**B**) and significant decreases in levels of HSP90 (**A**,**C**, but in F98 by SLCP) after treatment with Cur or SLCP treatments. The changes were more in the case of SLCP-treated cells in comparison to Cur-treated cells. Values are represented as mean ± SEM from two independent observations. * *p* < 0.05 and ** *p* < 0.01, in comparison to their respective vehicle-treated group.

CMA markers, such as HSC70, were unaltered in all three GBM cell lines, but LAMP2A levels were significantly down-regulated after treatment with Cur and or SLCP (Figure 5A,D,E). In contrast, HSP70 was increased in all three cell lines after treatment with Cur and or SLCP (Figure 5A,B), and the HSP90 level was also diminished in U-87MG and GL261, but was increased in F98 cells treated with SLCP (Figure 5A,C).

### 2.6. Cell Survival and Cell Death Markers Were also Modulated by SLCP More than Cur in GBM Cells.

**Figure 6:**
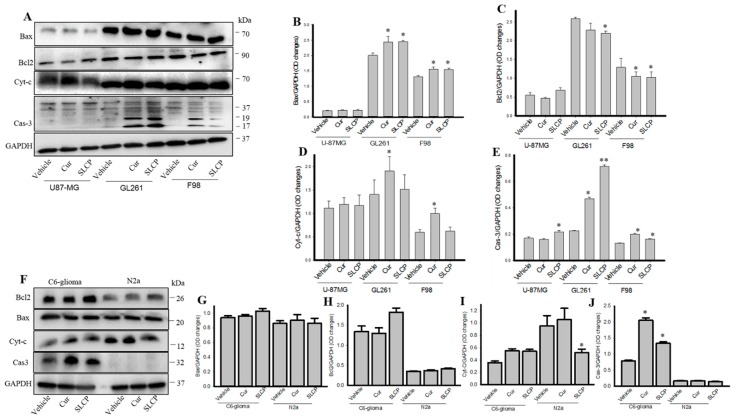
Cell death and cell survival markers in GBM cells. U-87MG, GL261, F98, C6-glioma, and N2a cells were treated with either Cur or SLCP (25 µM for 24-h) prior to Western blots analyses. Western blots analyses showed that SLCP and Cur treatments increased Bax, Cyt-c, and caspase-3 and decreased Bcl2 in all three GBM cell lines, in comparison to vehicle-treated cells (**A**–**E**). Cell survival and cell death markers were unaltered in the case of C6-glioma and N2a cells, except for Caspase-3, which was increased in C6-glioma after both of these treatments. Values are represented as mean ± SEM from two independent observations. * *p* < 0.05 and ** *p* < 0.01 in comparison to either in Cur-treated or to vehicle-treated cells.

There was a significant increase (*p* < 0.01) in Bax and Cyt-c and caspase-3 levels in both Cur and SLCP-treated cells in comparison to vehicle-treated cells (Figure 6A–E). In contrast, there was a significant decrease of Bcl-2 in GL261 and F98 cells (Figure 6A,B). Unlike GBM cell lines, Bax and Bcl2 were unaltered in C6-glioma and N2a cells (Figure 6F,G), but Cas-3 (Figure 6F,J) levels were increased in C6-glioma, while being unaltered in N2a cells after Cur or SLCP treatment, in comparison to vehicle-treated cells.

### 2.7. SLCP Treatment Produced Greater Increases in the Number of Autophagy Vacuoles in U-87MG Cells than Did Cur Treatments

**Figure 7:**
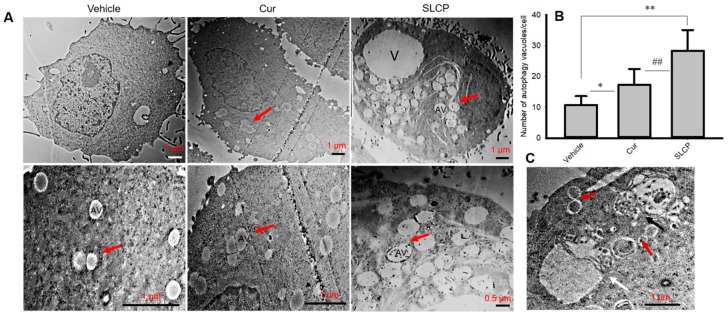
SLCP treatments resulted in more autophagy vacuole formations in U-87MG cells than did treatments with Cur. U-87MG cells were grown and treated with either Cur or SLCP (25 µM for 24-h). Cells were processed for TEM and images were taken with JEOL-TEM. (**A**): Representative TEM images showing that the numbers of autophagy vacuoles were significantly increased (**B**) in the case of SLCP-treated cells compared to Cur-treated cells. (**C**): Fusion of two autophagy vacuoles (red arrow), autophagolysosome complex (black arrow), and mitophagy (white arrow) after treatments with SLCP for 24 h. Scale bars indicate 1 µm. V-vacuole, AV-autophagy vacuoles, arrow indicates autophagy vacuoles. * *p* < 0.05 and ** *p* < 0.01 compared to vehicle group; ## *p* < 0.01 compared to Cur-treated cells.

To investigate the degree of formation of autophagy vacuoles (AV) after Cur or SLCP-treatment, U-87MG cells were treated with SLCP and/or Cur for 24 h. TEM images indicated that there were significantly more autophagy vacuoles in SLCP-treated cells (*p* < 0.01) in comparison to Cur-treated cells (Cur: 61.00% and in SLCP: 163.84%) (Figure 7A,B). In addition, SLCP treatments increased membrane blebbing, cytoskeleton disorientation, and chromosomal condensation more in U-87MG cells than Cur-treated cells (Figure S1).

## 3. Discussion

Accumulated experimental evidence supports the contention that the dysregulation of autophagy mechanisms significantly contributes to GBM cell death and survival [10]. In the present study, we compared the role of natural Cur or SLCP (a greater permeable solid lipid Cur formulation) on autophagy markers, including mitophagy and chaperone-mediated autophagy pathways in cultured GBM cells. We found a greater induction of autophagy markers, including an increased number of autophagy vacuoles, along with a significant decrease in the levels of mitophagy markers and inhibition of the PI3K-Akt/mTOR pathway by SLCP than by natural Cur in different GBM cell lines, without significantly affecting the rat glial tumor cells (C6-glioma) and mouse neuroblastoma (N2a) cells.

Given the present lack of effectiveness and the preponderance of side-effects when the current standard chemotherapies for GBM are used [28,29], many laboratories, including our own, have started investigating natural anti-cancer agents, such as Cur [2,30]. However, to avoid the poor solubility, rapid degradation, and limited bio-availability of natural Cur [17,23], we utilized more permeable solid lipid Cur particles (SLCP) [15,18,19]. Previously, we have demonstrated that SLCP has greater anti-cancer effects in U-87MG cells than does natural Cur [19]. However, its role in autophagy mechanisms in GBM cells remained unexplored. Given that Cur has been shown to induce autophagy, we hypothesized that using SLCP, with its higher solubility and greater membrane permeability, would have stronger modulatory effects on autophagy than natural Cur. Therefore, we investigated different autophagy markers in three different GBM cells (U-87MG, GL261, and F98) after treatment with Cur and or SLCP.

GBM cell metastasis can be prevented by induction of apoptosis [31]. However, cells can die by alternative pathways, such as autophagy. Interestingly, when autophagy is induced, either at earlier or later stages, it may lead to non-apoptotic cell death [32]. In addition, cells with a dysfunctional apoptotic pathway may undergo autophagic cell death [33]. Thus, the timing and magnitude of the cellular stresses seem to dictate whether autophagy or apoptosis will be activated. Moreover, autophagy preceded apoptosis, and this mechanism makes the cells more susceptible to death [10]. Given this, we sought to understand the mechanisms of autophagy, including mitochondrial autophagy (mitophagy) and CMA markers, in cultured GBM cells by comparing the treatment effects of SLCP with those of natural Cur.

To monitor the autophagy mechanism in GBM cells after treatment with Cur or SLCP, we investigated the Atg5, Atg7, Beclin-1 and LC3A/B, and p62. Atg5 and Atg7 are considered to be essential molecules for the induction of autophagy [34]. For example, defective Atg5 or Atg7 expression consequences decreased autophagic activity in animals [9]. In the present study, we observed an up-regulation of these two markers in all three GBM cells, (except Atg5 in U-87MG cells) (Figure 1) after treatment with Cur and or SLCP, suggesting the induction of autophagosome formation, which has been verified by transmission electron microscopic studies (Figure 7). Similarly, Beclin-1 is an important autophagosome initiation tumor suppressor protein, whose expression is reduced in many cancers. It interacts with Bcl-2 and can induce apoptosis by activating the function of mitochondrial permeability transition pore (MPTP) [35]. Overexpression of Beclin-1 in U-87MG cells enhances the capacity for cellular autophagy, whereas silencing of Beclin-1 decreases autophagic capacity [36]. We found an increase in beclin-1 levels after Cur and/or SLCP treatment, suggesting an enhancement of the autophagy pathway (Figure 1). Our findings are supported by Liang and colleagues, who also found that over-expression of Beclin-1 in U-87MG cells enhanced the capacity for cellular autophagy and induced apoptosis, whereas silencing of Beclin-1 decreased autophagic capacity [36].

LC3A/B is the key structural component of the autophagosome formation [37]. The amount of LC3-II reflects the number of autophagosomes and autophagy-related structures and a decrease of its level indicates an impairment of this process. Therefore, levels of LC3 are considered the most reliable marker to monitor autophagy. We observed an increase in the levels of LC3A/B-II after treatment with Cur or SLCP, indicating autophagosome formation was enhanced, which was supported by the recent in vitro work of Guo and colleagues [38]. Although the amount of LC3-II at a given time point does not necessarily estimate the autophagic activity, because inhibition of autophagosome degradation sometimes increases the amount of LC3-II, most cases reveal that increased amounts of LC3-II reflect the number of autophagosomes, thus increased levels of LC3A/B, suggesting that the autophagy mechanism was induced by these treatments (Figure 1). In addition, we also investigated the levels of sequestosome 1 (SQSTM1) or p62, which binds directly to LC3. Increased levels of LC3A/B-II and p62 indicate an increased accumulation of autophagosome formation, which is correlated with an increase in autophagic vacuoles (AV), as revealed by our TEM studies. Although increased levels of p62 inhibit autophagy mechanisms, and decreased levels can be observed when autophagy is induced, therefore, p62 levels may be used as a marker to study autophagic flux. We found that p62 levels were increased by Cur or SLCP treatment, which may be due to blocking of the fusion of autophagy vacuoles with lysosome or by the inhibition of a later maturation step of autophagosome degradation. The overall accumulated increase in AVs in cells by these treatments could induce autophagy-related cell death. Recently, Zanotto-Filho and colleagues showed that autophagy induction improves the efficacy of Cur and or TMZ combination therapy in animal models of glioblastomas, suggesting Cur is a potent autophagy inducer in GBM cells [39].

As GBM cells are resistant to apoptosis, the mammalian target for the rapamycin (mTOR) signaling pathway plays an important role [40]. In fact, the mTOR pathway has emerged as a major effector of cell growth and proliferation and is an attractive target for cancer therapy [41,42]. Indeed, mTOR and phosphorylated mTOR (p-mTOR, active form of mTOR) is a potent blocker of autophagy [43]. Thus, inhibiting the mTOR pathway could be a viable strategy to induce autophagy-related cell death to prevent GBM growth. These proteins are controlled by the cellular PI3K and Akt levels. Increases in PI3K activate Akt, which activates the mTOR pathway and induces tumorigenesis, whereas their inhibition prevents activation of this pathway [44]. We observed a significant decrease in levels of PI3Kp85, phosphorylated PI3Kp85, total Akt, p-Akt, mTOR, and p-mTOR after treatment with Cur and or SLCP, indicating PI3K-Akt/mTOR pathways were significantly affected by these treatments, which corresponds to recent findings of Yu and colleagues [45]. Decreased mTOR levels have been shown to induce autophagy [46]. Interestingly, we observed greater levels of PI3K-Akt/mTOR inhibition in SLCP-treated cells (Figure 4) than in Cur-treated cells, indicating greater induction of autophagy by SLCP than natural Cur, which was further supported by increased levels of autophagy markers and the number of AV (Figure 7). Recently, Guo and colleagues also reported that Cur may protect cells against oxidative stress-induced damage through induction of autophagy via inhibition of the Akt/mTOR pathway [38]. Similarly, using in vitro and in vivo models of GBM, Zhuang and colleagues also reported that Cur promotes differentiation of glioma-initiating cells by inducing autophagy [47]. Furthermore, Aoki and colleagues reported that Cur suppresses the growth of malignant gliomas in vitro and in vivo through induction of autophagy by inhibition of the Akt/mTOR/p70S6K pathway and activation of the ERK1/2 pathway [48]. The above observations were supported by the work of Zhao and colleagues, who reported that Cur potentiates the anti-tumor activities against GBM by suppressing the PI3K/AKT and NF-κB/COX-2 signaling pathways [14]. All these previous observations further supported and confirmed our findings.

In addition, we investigated the mitophagy markers. Mitophagy is a selective form of macroautophagy in which mitochondria are targeted for degradation in autophagolysosomes [49,50]. Although mitophagy is not a mechanism of autophagy, it is a special type of autophagy pathway, but it has beneficial effects, especially for the elimination of old and/or damaged mitochondria, thus maintaining the integrity of the mitochondrial pool. Inhibition of mitophagy has adverse effects on mitochondrial health and cell survival [49]. In addition, mitophagy also plays a key role in reducing mitochondrial mass [49]. We have investigated mitophagy markers, such as BNIP3L/NIX, FUNDC1, BNIP3, PINK-1, and HIF-1α (Figure 3). BNIP3L (Bcl2 and adenovirus E1B 19-kDa-interacting protein 3-like), also known as NIX, are the proteins which interact with Bcl2 and are involved in cell death and autophagy, suggesting that BNIP3L/NIX are implicated in the pathogenesis of cancer [51]. BNIP3L is required for interaction with Bcl2, the main anti-apoptotic protein present in mitochondria, whereas decreased levels of BNIP3L may inhibit interactions with Bcl2, thus indirectly inducing cell death [51]. Similarly, BNIP3 is another mitophagy marker, which has a similar role in mitophagy, like BNIP3L/NIX. We found a greater decrease in levels of both NIX and BNIP3 in the case of SLCP (Figure 3), which suggests that SLCP has greater capability to induce cell death. Moreover, in tumor cells, NIX and BNIP3 regulate mitophagy in response to hypoxia, and the deregulation of NIX and BNIP3 expression is associated with increased tumor growth [51]. One of the factors noted in most malignancies is the hypoxic environment, where hypoxia-inducing factor-1α (HIF-1α) levels increase. This factor is a positive regulator of NIX and BNIP3 expression. Therefore, reduction of HIF-1α may down-regulate NIX or BNIP3 levels. For example, knock-down of HIF-1α in glioma cells significantly impairs their migration in vitro, as well as their ability to invade into the brain parenchyma in vivo [52]. In addition, HIF-1α acts as an activator of angiogenic factors, such as placenta-like growth factor and platelet-derived growth factor. Therefore, decreased levels of HIF-1α may be one of the reasons for the down-regulation of NIX or BNIP3 levels, indicating a reduction of mitophagy and induction of cell death.

Like NIX and BNIP3, FUNDC1 is an adaptor molecule present at the outer membrane of mitochondria [53]. NIX, BNIP3, and FUNDC1 interact with Bcl-2 or Bcl-XL and modulate in binding with LC3 and recruit components of the autophagy machinery to the mitochondria. Therefore, inhibition of these adaptor proteins, along with decreased HIF-1α after treatment with Cur and or SLCP, indicates decreased mitophagy, which may lead to induction of cell death [49]. Greater inhibition of these proteins by SLCP treatment in comparison to Cur indicates SLCP is a stronger negative regulator on these proteins, which suggests that a greater amount of Cur is required to inhibit mitophagy, as well as induction of cell death. Given that mitophagy may also be regulated by PINK-1/Parkin, we investigated the levels of PINK-1 after treatment with Cur and or SLCP. We found a significant reduction of PINK-1 levels (Figure 3), suggesting Cur- and or SLCP-induced mitophagy is PINK-1 dependent. Other than PINK-1, mitophagy may also be regulated by the Parkin pathway, but the role of Parkin in the regulation of cell death is debated. Importantly, PINK1 shuttles between the cytosol and mitochondria in healthy cells. It plays a vital role in communicating the collapse of the mitochondrial membrane potential and it can stabilize on the outer membrane of depolarized mitochondria and recruit Parkin, which is initially inactive. In addition, PINK1 can phosphorylate Parkin on the ubiquitin-like (UBL) domain, resulting in an increase of its ubiquitin ligase activity and the formation of polyubiquitin chains on the surface of depolarized mitochondrial membranes, which could act as a Parkin activator by overcoming the autoinhibitory mechanism of Parkin. Therefore, we investigated the levels of PINK1 rather than Parkin.

In addition to macroautophagy and microautophagy, we also investigated the status of chaperone-mediated autophagy (CMA), which is a special type of autophagy for degradation of tiny proteins aggregates. It requires chaperones, such as heat shock cognate 70 (HSC70) and lysosome-associated membrane protein type 2A (LAMP-2A). Although HSC70 levels were unaltered, LAMP2A was significantly down regulated by Cur or SLCP, indicating CMA was also inhibited (Figure 5). In addition, we also checked whether the other chaperones, such as HSP70 and HSP90, were affected by Cur and or SLCP treatments in GBM cells. We observed that there was an upregulation of HSP70, whereas opposite effects were observed in the case of HSP90 in all these GBM cells (except F98 cells) after SLCP treatment. HSP90 become upregulated in cancer and the inhibition of HSP90 by Cur or SLCP suggested that Cur/SLCP acts as a HSP90-inhibitor [54].

Previously, we have confirmed that the SLCP induced greater cell death in U-87MG cells by using MTT, TUNEL, Annexin-V staining, and comet assays [19]. In support of our previous findings, we presently observed more membrane blebbing, actin or cytoarchitectural damage, and chromosomal condensation (Figure S1), as well as induction of cell-death related proteins and reduction of cell survival proteins (Figure 6) in the case of SLCP, when compared to Cur-treated cells. This finding indicates that a higher amount of Cur is required to damage the cells, which can be achieved more efficiently by SLCP treatments. Moreover, an increased number of autophagy vacuoles (Figure 7), chromosomal condensation, and membrane blebbing, as seen by TEM images (Figure S1), confirmed that SLCP can induce autophagy and cell death more efficiently than by using Cur (Figure 6). Extrapolating these results, we have assessed that Cur and SLCP have roles on autophagy mechanisms in cultured GBM cells, without affecting the rat glial tumor cell line (C6-glioma) or mouse neuroblastoma cell line (N2a cells). To this end, the C6-glioma and N2a cells that were treated with the same concentrations (25 µM) of Cur and/or SLCP consistently revealed the lowest levels of autophagy and cell death markers (Figure 2 and Figure 6), which suggests that Cur and SLCP can specifically target its therapeutic effects on GBM cells (Type-IV glioma), rather than glial tumor or neuroblastoma cells. In addition, during cellular stress, pro-survival and pro-death processes are concomitantly activated, which depends on the degree of stress. Mild cellular stress causes damage to a few mitochondria, which are rapidly sequestered by autophagosomes, whereas severe stress induces mitochondrial damage and autophagy is unable to efficiently clear this. During these circumstances, mitochondria release pro-death proteins, such as cytochrome c, apoptosis inducing factor (AIF) and SMAC/Diablo, that can activate the cell death pathway. As treatment of Cur/SLCP induced cellular stress, therefore, it can induce mitochondrial damage and activate the cell death pathway. Overall, Cur or SLCP treatment induced cellular stress, which interfered in autophagy, mitophagy, and the cell death and survival pathway as observed in cultured GBM cells.

As autophagy determines cell death and survivability, therefore, the role of autophagy in GBM cell death and survivability after Cur and SLCP treatment needs to be critically analyzed. In fact, not only under disease conditions, but even under normal physiological conditions, the autophagy mechanism is active and can promote cell death or increase cell growth. For example, nutrient starvation, deprivation of supporting factors, or a hypoxic environment increases cell survivability [55,56]. Similarly, autophagy-dependent program cell death also occurs during mammalian embryogenesis [57] and in the case of apoptosis-resistant cells, such as in the absence of the pro-apoptotic proteins, Bax and Bak [58]. Moreover, many cellular environment autophagy cross-talks with the apoptotic machineries and directly inhibits apoptosis [59]. Therefore, the connections between autophagy and cell death are very complicated and controversial. In the case of cancer cells, autophagy may act as a tumor suppressor, as well as inducing tumor growth [60]. Some reports suggest that induction of autophagy can suppress tumor growth, whereas its prolonged activation may kill cancer cells with a high apoptotic threshold. In contrast, prolonged inhibition of autophagy may lead to cell survival instead of cell death. Most interestingly, the apoptotic pathways become mutated in human tumors, where autophagy plays alternative forms of PCD to prevent their growth. In our study, we found an induction of autophagy by Cur and or SLCP treatment, but whether autophagy was the mechanism by which GBM cells were dying (cell death by autophagy) or autophagy was present during cell death (cell death with autophagy) is an open question. As we know, when cell death is mediated by autophagy and if the cell death is prevented after inhibition of autophagy, then it is considered as “autophagic cell death”. In our study, we did not investigate the cell death after inhibition of the autophagy mechanism, therefore, we were not sure whether it was a “autophagic cell death”. However, the “autophagic cell death” is also characterized by the presence of abundant autophagosomes and lack of phagocytic activities, and we found many autophagosomes in U-87MG cells after treatment with Cur and or SLCP (Figure 7), which suggests that Cur and or SLCP may have partially induced cell death caused by autophagy, along with apoptotic death [19]. However, we prefer to describe this phenomenon as “cell death with autophagy” rather than an important effector mechanism of cell death because we do not have concrete evidence to confirm whether the cell death was caused by autophagy. Most importantly, Cur and/or SLCP treatment caused DNA damage [19], and disruption of the cytoskeleton (Figure S1), which induced autophagy and suppress tumor growth, suggesting that these treatments have a negative impact on GBM cell survivability. Therefore, further experiments are needed for a better understanding of the role of autophagy mechanisms on GBM cell death and survival after treatment with Cur and or SLCP in order to apply its beneficial role for GBM therapy.

## 4. Materials and Methods

### 4.1. Chemicals

Curcumin (Purity >65%; catalog no: C1386-50G (Sigma, St. Louis, MO, USA), propidium iodide (PI), and other accessory chemicals were procured from Sigma (St. Louis, MO, USA). Hoechst 33342 was purchased from ThermoFisher Scientific (Grand Island, NY, USA). Solid lipid particles containing Cur (SLCP or Longvida, which contains 26% pure Cur) were gifted from Verdure Sciences (Noblesville, IN, USA). The SLCP has been well characterized by us and others in collaboration with Verdure Sciences, including clinical studies in Alzheimer’s disease [19,27]. The human origin GBM cell line (U-87MG; catalog no: HTB-14), rat GBM cells (F98, catalog no: ATCC^®^ CRL2397™), mouse glioma (C6-glioma, catalog no: ATCC^®^ CCL107™), and N2a (catalog no: ATCC^®^ CCL-131™) cells were purchased from ATCC (Manassas, VA, USA), whereas the mouse GBM cell line (GL261) was procured from DCTD/DTP Tumor Repository at the National Cancer Institute. All the antibodies used in this study are documented in Table 1.

### 4.2. Cell Culture

U-87MG and N2a cells were grown with Eagle’s Minimum Essential Medium (EMEM, GIBCO) containing 10% heat-inactivated fetal bovine serum (FBS), and penicillin/streptomycin (100 IU/mL penicillin and 100 µg streptomycin/mL). The Gl261 cells were cultured in Roswell Park Memorial Institute Medium-1640 (RPMI-1640), along with 10% FBS and pen/strep (100 IU/mL penicillin and 100 µg streptomycin/mL), and F98 cells were grown on Dulbeco’s Modified Eagle’s Medium (DMEM), along with 10% FBS and pen/strep, (100 IU/mL penicillin and 100 µg streptomycin/mL). The C6-glioma cells were grown in F12K media along with 2.5% FBS and 15% horse serum and 100 IU/mL penicillin and 100 µg streptomycin/mL. The cultures were maintained at 37 °C in a humidified atmosphere at 5% CO_2_. Prior to the experiment, the cells were grown either in a 75 cm^2^ culture flask, or on glass cover slips, with fresh media and antibiotics, but without growth factors, depending on the experimental setup.

### 4.3. Curcumin and or SLCP Treatment

Cur was solubilized in pure methanol (100%), as described previously (28), and then diluted in the Hank’s balanced salt solution (HBSS) to obtain its desired concentration before being added to the culture flask containing the cells. The final methanol concentration was ≤0.1% and the same amount of methanol was added to the vehicle-treated cell. The final Cur or SLCP concentration was 25 µM. This dose was selected on the basis of our dose dependent cell viability data (Figure S1) [34].

### 4.4. Immunocytochemistry and Confocal Imaging of Autophagy Markers.

Immunocytochemistry of Atg5, Atg7, Beclin-1, and LC3A/B were performed as described previously [24]. Briefly, U-87MG and N2a cells were grown (1 × 10^5^/well) on a Petri-plate containing glass cover slips in EMEM with pen/strep (100 IU/mL penicillin and 100 µg streptomycin/mL) for 24 h and then treated with Cur and/or SLCP (25 µM) for another 24 h. Then, the cells were fixed with 4% paraformaldehyde after washing with cold PBS (0.1 mM, pH 7.4) and incubated with rabbit anti-Atg5, Atg7, Beclin-1, and LC3A/B monoclonal and polyclonal antibodies (1:200, see Table 1) overnight at 4 °C, followed by incubation with the respective secondary antibodies (1:500) tagged with Alexa-fluorophore 560 (Molecular Probes, OR) for 1 h at room temperature. Nuclei were stained with Hoechst 33342 (20 mM, ThermoFisher Scientific, Grand Island, NY, USA) for 5 min and visualized using a table top Fluoview confocal laser scanning microscope (FV1oi, Olympus) using appropriate filters for excitation and emission.

### 4.5. Transmission Electron Microscopy (TEM)

U-87MG cells were processed for TEM as described by Schrand and colleagues [61]. Briefly, U-87MG cells were grown in 60 mm Petri plate (~10^6^ cells/mL) in EMEM with pen/strep (100 IU/mL penicillin and 100 µg streptomycin/mL) for 24 h. The next day, the cells were treated with Cur and or SLCP (25 µM) for 24 h. After treatment, the cells were thoroughly rinsed with fresh serum free media at room temperature (RT) for 3 times, 5 min each. Then, the cells were treated with 0.25% trypsin-EDTA solution for 1–2 min and the cell suspension was taken in a 15 mL conical tube and centrifuged for 5 min at 1000× *g* at room temperature. Supernatant was removed and 1 mL of fresh 2.5% glutaraldehyde/formaldehyde (dissolved in 0.1 mM PBS, pH 7.4) was added and kept for 2 h at RT. After fixation, the cells were thoroughly rinse with PBS, three times for 10 min each, and 1 mL of 1% osmium tetroxide (dissolved in PBS) was added and allowed for 1 h at RT. Then, the cell pellet was rinsed in PBS five times for 10 min each and then washed in double-distilled water (ddH_2_O) two times for 10 min each. After centrifugation, the cell pellet was dehydrated through a graded series of ethanol concentrations (50%, 70%, 90%, and 100%) for 10 min each. The cells were treated with propylene oxide: ethanol mixture (1:1) for 30–45 min, then this mixture was replaced with 100% propylene oxide for 10 min, followed by a propylene oxide: resin (1:1) for 45 min before continuing in 100% resin overnight. The sample block was prepared with small plastic cubes at the flat face and cells reached the bottom of the capsule. For sectioning, stereomicroscope lenses were adjusted to the lowest magnification and the lighting was set to focus on the sample. A glass knife was inserted into the knife holder and positioned near the sample block face in the ultramicrotome. The block was trimmed manually by advancing the glass knife attached to the ultra-microtome while viewing the sample through the stereomicroscope lenses. An 80-nm thick section was made, and several sections were collected onto a TEM grid (300-mesh Cu, with support film such as formvar/carbon) and sections on grids were allowed to dry for a few minutes, then carefully placed in a grid storage box using fine-tipped tweezers. For staining, a piece of parafilm was placed in a glass petri dish, onto which a few drops of water or stain were added. Then, the grid was placed face down on a drop of ddH_2_O for 1–2 min. Then, the grid was transferred face down onto a drop of 1% uranyl acetate and lead acetate (filtered through a 0.2-μm syringe filter) for 30 min, followed by the grid being dipped into a drop of double distilled H_2_O to rinse. Then, the grids were blot dried using Whatman’s filter paper and it was placed in a grid box for storage until imaging. At least 10 individual cells were imaged from each group and the number of autophagy vacuoles were counted from each of the cells manually.

### 4.6. Western Blot

To investigate different autophagy markers, Western blot was performed as described previously [19,24]. Briefly, after the stipulated period of each experiment, the media was removed and U-87MG, GL261, and F98 cells were washed with cold PBS, scrapped, collected in Eppendorf tubes, centrifuged, and pellets were lysed with cold radio-immunoprecipitation assay (RIPA) buffer, along with protease and phosphatase inhibitors. Total protein was measured from each of the samples by Pierce protein assay reagent. Equal amounts of protein, per lane, were loaded and electrophoresed on 10% Tris-glycine gel and transferred to PVDF membrane (Millipore, Bedford, MA). After probing with respective primary (1:1000; see Table 1) and secondary antibodies, the blots were developed with Immobilon^TM^ Western Chemiluminescent HRP-substrate (Millipore, Billeria, MA). The images were taken by a gel documentation system (Bio-Rad) with an automated exposure time. The relative optical density (OD) was measured using Image-J software (https://imagej.nih.gov/ij/). To ensure equal protein loading in each lane, the blots were probed with either β-tubulin or GAPDH.

### 4.7. Statistical Analysis

The data were expressed as mean ± SEM. Data were analyzed using one-way analysis of variance (ANOVA), followed by post-hoc Tukey HSD (honestly significant difference) test. Probability ≤0.05 was considered as statistically significant.

## 5. Conclusions

Overall, we demonstrated that Cur and or SLCP treatment induced autophagy and reduced mitophagy, probably through inhibition of the PI3K-Akt/mTOR signaling pathway in cultured GBM cells. In addition, cell death markers were induced, and cell survival markers were down-regulated by Cur and or SLCP. Importantly, SLCP showed greater induction of autophagy and greater inhibition of mitophagy markers, along with greater disruption of the PI3K-Akt/mTOR signaling pathway than Cur. There were lowest treatment effects on autophagy and mitophagy in rat glial tumor cells and mouse neuroblastoma cells. Therefore, the data presented demonstrated that induction of autophagy and reduction of mitophagy by Cur and or SLCP treatment, suggesting that treatments with SLCP to prevent GBM cell growth and proliferation may have promising clinical utility.

## Figures and Tables

**Table 1:** Sources of different antibodies used in this study.

Antibodies	Source	Type	Company	Catalog No.	Address
Atg5	Rabbit	Monoclonal	Cell signaling Technology	12994S	Danvers, MA, USA
Atg7	Rabbit	Monoclonal	Cell signaling Technology	8558S	Danvers, MA, USA
Beclin-1	Rabbit	Polyclonal	Cell signaling Technology	3738S	Danvers, MA, USA
LC3A/B	Rabbit	Polyclonal	Cell signaling Technology	4108S	Danvers, MA, USA
p62	Rabbit	Polyclonal	Cell signaling Technology	5114S	Danvers, MA, USA
mTOR	Rabbit	Polyclonal	Cell signaling Technology	2972S	Danvers, MA, USA
p-mTOR	Rabbit	Monoclonal	Cell signaling Technology	2971S	Danvers, MA, USA
PI3Kp85	Rabbit	Monoclonal	Cell signaling Technology	4292S	Danvers, MA, USA
BNIP3L/NIX	Rabbit	Monoclonal	Cell signaling Technology	12396S	Danvers, MA, USA
FUNDC1	Rabbit	Monoclonal	EMD Millipore	ABC506	Burlington, MA, USA
HIF-1α	Rabbit	Monoclonal	Cell signaling Technology	14179S	Danvers, MA, USA
PINK-1	Rabbit	Monoclonal	Cell signaling Technology	6946S	Danvers, MA, USA
Cyt-c	Rabbit	Monoclonal	Cell Signaling Technology	4272S	Danvers, MA, USA
Caspase-3	Rabbit	Monoclonal	Cell Signaling Technology	9661S	Danvers, MA, USA
Bax	Rabbit	Polyclonal	Cell signaling Technology	2772S	Danvers, MA, USA
Bcl-2	Mouse	Monoclonal	Santa Cruz Biotech	Sc-7382	Santa Cruz, CA, USA
Akt	Rabbit	Monoclonal	Cell signaling Technology	9272S	Danvers, MA, USA
pAkt (Ser473)	Rabbit	Monoclonal	Cell signaling Technology	4060S	Danvers, MA, USA
HSP70	Rabbit	Polyclonal	Cell signaling Technology	4872S	Danvers, MA, USA
HSP90	Rabbit	Polyclonal	Cell signaling Technology	4877S	Danvers, MA, USA
HSC70	Rabbit	Polyclonal	Cell signaling Technology	8444S	Danvers, MA, USA
LAMP2	Mouse	Polyclonal	Santa Cruz Biotech	sc-20004	Santa Cruz, CA, USA
GAPDH	Rabbit	Monoclonal	Cell signaling Technology	2118S	Danvers, MA, USA
β-tubulin	Rabbit	Monoclonal	Cell signaling Technology	2146S	Danvers, MA, USA

## Supplementary Materials

Supplementary materials can be found at http://www.mdpi.com/1422-0067/20/2/399/s1. Figure S1: SLCP induced greater changes of membrane, cytoskeleton, and nuclear morphology than Cur in U-87MG cells.

## Abbreviations

GBM	Glioblastoma
SLCP	Solid lipid curcumin particles
Cur	Curcumin
Atg	Autophagy-related protein
Akt	Protein kinase B
PI3K	Phosphatidyl inositol-3 kinase
mTOR	Mechanistic target of rapamycin
HSP	Heat shock protein
TMZ	Temozolomide
PCD	Program cell death
ATP	Adenosine triphosphate
SLPs	Solid lipid particles
AD	Alzheimer’s disease
PI	Propidium iodide
EMEM	Eagle’s Minimum Essential Medium,
FBS	Fetal bovine serum
HBSS	Hank’s balanced salt solution
DMEM	Dulbecco’s Modified Eagle’s Medium
FITC	Fluorescent isothiocyanate
DPBS	Dulbecco’s phosphate buffer saline
ROS	Reactive oxygen species
BNIP3	Bcl-2/adenovirus E1B 19 kDa-interacting protein
Bax	Bcl_2_-associated X protein
Bcl2	B-cell lymphoma 2
NF-kB	Nuclear factor kappa beta
ATCC	American type cell culture
SEM	Standard error of mean
PBS	Phosphate buffer saline
EDTA	Ethylene-di-amino-tetra-acetic-acid
RPM	Revolution per minute
mM	Millimolar
RIPA	Radio immunoprecipitation assay
SDS	Sodium dodecyl sulfate
BCA	Bicinchoninic acid assay
PVDF	Polyvinylidene fluoride
ANOVA	One-way analysis of variance
HSD	Honestly significant difference
µM	Micromolar
AU	Arbitrary unit
OD	Optical density
SDS-PAGE	Sodium dodecyl sulfate polyacrylamide gel electrophoresis
TBS	Tris buffer saline
CMA	Chaperone-mediated autophagy
HSC70	Heat shock cognate 70
LAMP-2A	Lysosome-associated membrane protein type 2A
G2/M	Gap2/mitosis
VEGF	Vascular endothelial growth factor
