# Supplementary material for: Curcumin and Solid Lipid Curcumin Particles Induce Autophagy, but Inhibit Mitophagy and the PI3K-Akt/mTOR Pathway in Cultured Glioblastoma Cells
Source: Int J Mol Sci. 2019 Jan 18;20(2):399. doi: 10.3390/ijms20020399 (PMC6359162; doi:10.3390/ijms20020399)
Supplement: Supplementary file 1 [file ijms-20-00399-s001.pdf]

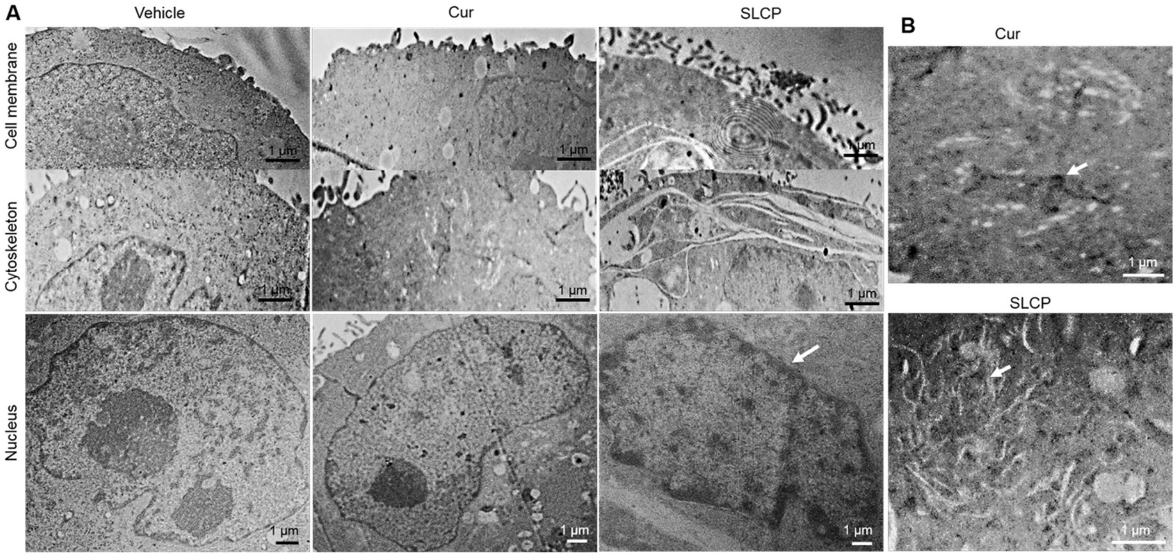

Figure S1. SLCP induced greater changes of membrane, cytoskeleton, and nuclear morphology than Cur in U-87MG cells. U-87MG cells were grown in EMEM, along with 100 IU/mL penicillin and 100 μg streptomycin/ml for 24 h and then treated with either Cur or SLCP (25 μM) for 24-h. Cells were processed for TEM and images were taken with JEOL-TEM. **A**: Representative TEM images showing that there was an increased membrane blebbing, cytoskeletal disorientation, and chromosomal condensation in the case of SLCP-treated cells in comparison to Cur or vehicle-treated cells. **B**: Actin filaments were degenerated by SLCP more than Cur-treated cells.
